# Pre-treatment MRI minimum apparent diffusion coefficient value is a potential prognostic imaging biomarker in cervical cancer patients treated with definitive chemoradiation

**DOI:** 10.1186/s12885-016-2619-0

**Published:** 2016-07-28

**Authors:** Daniel Grossi Marconi, Jose Humberto Tavares Guerreiro Fregnani, Rodrigo Ribeiro Rossini, Ana Karina Borges Junqueira Netto, Fabiano Rubião Lucchesi, Audrey Tieko Tsunoda, Mitchell Kamrava

**Affiliations:** 1 Department of Radiation Oncology, Barretos Cancer Hospital, Antenor Duarte Villela, 1331, Barretos, Sao Paulo 14784-400 Brazil; 2 Department of Gynecology Oncology, Barretos Cancer Hospital, Barretos, Sao Paulo Brazil; 3 Department of Radiology, Barretos Cancer Hospital, Barretos, Sao Paulo Brazil; 4 Department of Radiation Oncology, University of California Los Angeles, Los Angeles, CA USA

**Keywords:** Cervical cancer, Diffusion weighted imaging, Chemoradiation, MRI

## Abstract

**Background:**

Diffusion Weighted (DW) Magnetic Resonance Imaging (MRI) has been studed in several cancers including cervical cancer. This study was designed to investigate the association of DW-MRI parameters with baseline clinical features and clinical outcomes (local regional control (LRC), disease free survival (DFS) and disease specific survival (DSS)) in cervical cancer patients treated with definitive chemoradiation.

**Methods:**

This was a retrospective study approved by an institutional review board that included 66 women with cervical cancer treated with definitive chemoradiation who underwent pre-treatment MRI at our institution between 2012 and 2013. A region of interest (ROI) was manually drawn by one of three radiologists with experience in pelvic imaging on a single axial CT slice encompassing the widest diameter of the cervical tumor while excluding areas of necrosis. The following apparent diffusion coefficient (ADC) values (×10^−3^ mm^2^/s) were extracted for each ROI: Minimum - ADC_min_, Maximum - ADC_max_, Mean - ADC_mean_, and Standard Deviation of the ADC - ADC_dev_. Receiver operating characteristic (ROC) curves were built to choose the most accurate cut off value for each ADC value. Correlation between imaging metrics and baseline clinical features were evaluated using the Mann Whitney test. Confirmatory multi-variate Cox modeling was used to test associations with LRC (adjusted by gross tumor volume – GTV), DFS and DSS (both adjusted by FIGO stage). Kaplan Meyer curves were built for DFS and DSS. A *p*-value < 0.05 was considered significant.

Women median age was 52 years (range 23–90). 67 % had FIGO stage I-II disease while 33 % had FIGO stage III-IV disease. Eighty-two percent had squamous cell cancer. Eighty-eight percent received concurrent cisplatin chemotherapy with radiation. Median EQD2 of external beam and brachytherapy was 82.2 Gy (range 74–84).

**Results:**

Women with disease staged III-IV (FIGO) had significantly higher mean ADC_max_ values compared with those with stage I-II (1.806 (0.4) vs 1.485 (0.4), *p* = 0.01). Patients with imaging defined positive nodes also had significantly higher mean (±SD) ADC_max_ values compared with lymph node negative patients (1.995 (0.3) vs 1.551 (0.5), *p* = 0.03).

With a median follow-up of 32 months (range 5–43) 11 patients (17 %) have developed recurrent disease and 8 (12 %) have died because of cervical cancer. ROC curves based on DSS showed optimal cutoffs for ADC_min_ (0.488 × 10^−3^), ADC_mean_ (0.827 × 10^−3^), ADC_max_ (1.838 × 10^−3^) and ADC_dev_ (0.148 × 10^−3^). ADC_min_ higher than the cutoff was significantly associated with worse DFS (HR = 3.632–95 % CI: 1.094–12.054; *p* = 0.035) and DSS (HR = 4.401–95 % CI: 1.048–18.483; *p* = 0.043).

**Conclusion:**

Pre-treatment ADC_max_ measured in the primary tumor may be associated with FIGO stage and lymph node status. Pre-treatment ADC_min_ may be a prognostic factor associated with disease-free survival and disease-specific survival in cervical cancer patients treated with definitive chemoradiation. Prospective validation of these findings is currently ongoing.

## Background

In Brazil, it is estimated that 18,500 women are diagnosed with cervical cancer annually, and 8,400 die [1]. While screening rates for cervical cancer have improved in many countries, there are still a significant number of women who present with locally advanced disease that will require definitive treatment with chemoradiation. Advances in image-guided brachytherapy using Magnetic Resonance Imaging/planning (MRI) rather than 2-dimmensional techniques is significantly improving the outcomes, and changing their patterns of recurrence [2]. With image-based brachytherapy the vast majority of these patients are achieving local control of their tumors with limited serious acute or late morbidity. More women are now recurring with distant rather than local failures with marginal outcomes with systemic therapy in these cases [3]. Efforts are now underway on the OUTBACK trial [4], for example, to potentially improve these clinical outcomes with the addition of systemic chemotherapy following the completion of definitive chemoradiation. One of the challenges of this approach is being able to identify patients at highest risk for poor outcomes following chemoradiation alone. Advances in functional imaging with Positron Emission Tomography (PET) and quantification of a standardized uptake value (SUV) can provide prognostic information that may be helpful in identifying women populations at higher risk of failure, thereby allowing for an enriched patient population that is more likely to benefit from escalated therapy [5–7].

In low-middle income countries there are a limited number of cyclotrons available for PET imaging making it impossible to integrate this technology into the routine management of cervical cancer patients. MRI is, however, readily available and routinely utilized for cervical cancer staging in many countries but standard imaging sequences only provide anatomical, and not functional, information. Newer MRI sequences such as diffusion-weighted imaging (DWI) provide functional information by characterizing the diffusion of water between cells [8–10]. This can be quantitated, similar to an SUV on PET scans, by calculating an apparent diffusion coefficient (ADC) value. Previous investigators have demonstrated high concordance of tumor sub volumes with increased metabolic activity on PET with increased cellular density on DWI imaging suggesting ADC values may have similar prognostic value as SUV_max_ [8, 11].

DWI imaging has previously been studied in cervical cancer patients with mixed results regarding its utilization as a prognostic/predictive marker [12–14, 15–18]. Given the variation in correlations with DWI imaging, we investigated whether baseline MRI DWI imaging features correlate with clinical outcomes in women with locally advanced cervical cancer treated with definitive chemoradiation.

## Methods

This was a retrospective study of cervical cancer women treated at Barretos Cancer Hospital, approved by the Research Ethics Committee. Patients were treated using radiation therapy with or without concurrent chemotherapy and who had an MRI of the pelvis performed prior to the start of treatment between January 2012 and March of 2013. A total of 135 patients were identified. Forty-six were excluded from further analysis because either their MRI was not performed at our institution or diffusion weighted imaging was not performed. Ten additional women were excluded because they were treated with palliative intent and 13 more were excluded because they did not have sufficient clinical follow-up information available. This left 66 women available for complete analysis.

The study group was classified according to the revised 2009 FIGO staging system. The extent of tumor involvement was based on both clinical examination and MRI findings (i.e. a patient with parametrial involvement on clinical examination but clear involvement on MRI imaging to the pelvic sidewall was classified as FIGO Stage IIIB). Positive lymph nodes were based on MRI findings. A lymph node was considered positive if it had a maximum diameter larger than 1 cm with heterogeneity of signal or an irregular contour. Enlarged lymph nodes were not routinely pathologically confirmed.

Eight patients received radiation alone and 58 were treated with chemoradiation. All 66 women received high dose rate (HDR) brachytherapy as a component of their treatment. The mean (SD) external beam and HDR doses were: 44.92 (0.62) Gy and 27.05 (1.67) Gy (7 Gy × 4 being the most common fractionation), respectively. The mean (SD) equivalent dose in 2-Gy fractions (EQD2) for external beam plus HDR-brachytherapy was 82.2 (2.8) Gy. There was no lymph node boost.

Radiation was delivered using a standard linear accelerator with either 6MV or 15MV beams and planned using a 3D planning method with organ at risk and target volumes contoured by a radiation oncologist. Intensity modulated radiation therapy was not used. All patients were planned using Eclipse version 8.0 (Varian Medical Systems, Palo Alto, CA, USA).

For the brachytherapy, four fractions of HDR using a tandem and ovoid applicator were delivered to all patients (except two that only received three fractions of 7 Gy). The dose was prescribed to point A and was planned based on 2-dimmensional films. Bladder and rectal points were placed as per ICRU 38 guidelines. The constraints for a prescription of 7 Gy were 71 % (bladder) and 58 % (rectum). Dose prescription was diminished to 6.5 Gy or 6 Gy when the constraints were extrapolated. Brachytherapy planning was performed using GammaMed™ (Varian Medical Systems Inc., Palo Alto, CA, USA).

The majority of women (82 %) were treated with concurrent cisplatin chemotherapy at a dose of 40 mg/m^2^ weekly. Four women received concurrent carboplatin and eight did not receive concurrent chemotherapy.

All patients were followed up with physical exam, abdominal/pelvic imaging and surveillance pap smears every 3 to 6 months.

Disease recurrence was determined radiographically by RECIST 1.1 criteria [19] and was not pathologically confirmed. Local regional control was defined as the time from biopsy to local (uterine cervix or vagina) or regional recurrence (pelvic lymph node). Disease free survival (DFS) was defined as the time from biopsy to tumor progression. Disease specific survival (DSS) was defined as the time between biopsy to death by cancer.

### Imaging technique and analysis

All images were performed at baseline assessment (before any treatment) on one of two scanners: Achieva 3.0 Tesla, Philips Healthcare, Netherlands or a Signa HDX’T 1.5 Tesla, GE Healthcare, Milwaukee. All patients had trans-vaginal ultrasound gel administered prior to the start of their MRI. The sequences acquired included T2-weighted sequences of the whole pelvis and abdominal region below the renal arteries, axial T1-weighted sequences of the whole pelvis, T2-weighted sequences in the sagittal, axial and coronal planes at an angle through the plane of the cervix, and diffusion-weighted sequences. For the diffusion sequence, the field of view was 40 × 40, the matrix size was 512, with b-values of 0, 600 (3 T scanner) and 0, 800 (1.5 T scanner). Acquisition time was 6 min. Voxel size was 2.34 mm (RL), 3.19 mm (AP). Repetition time was 1800 ms and slice thickness was 3 mm.

**Fig. 1:**
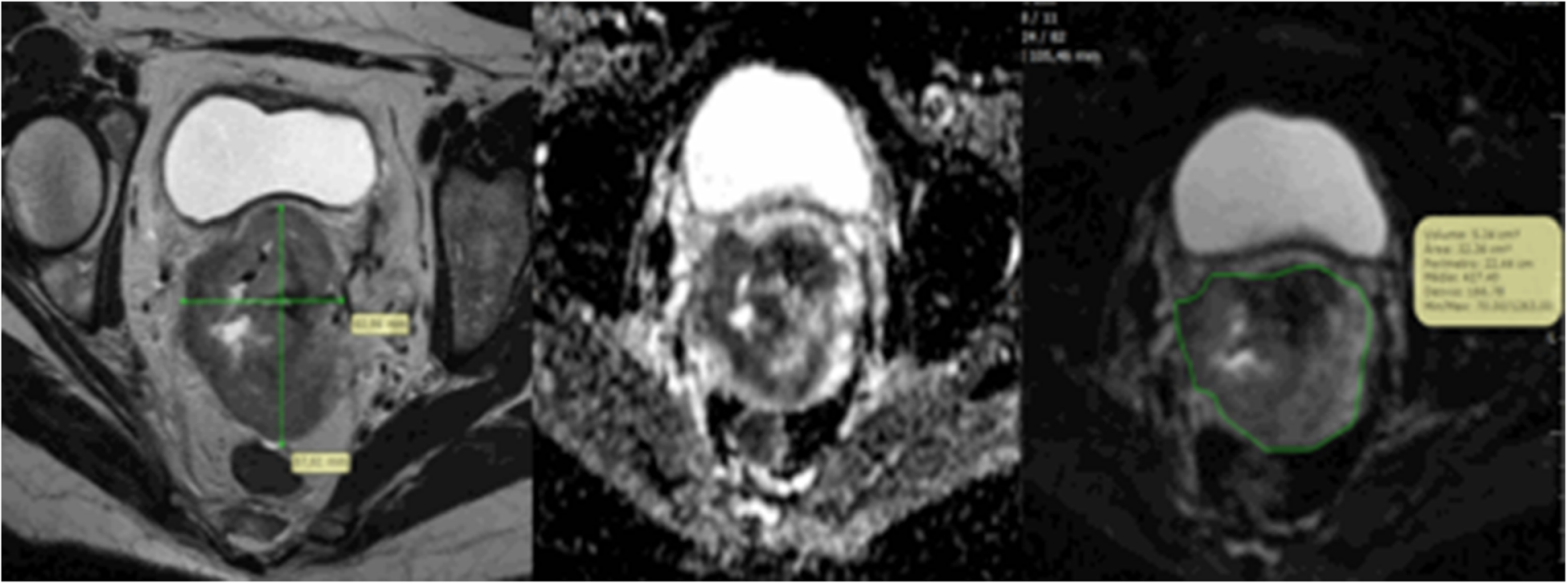
Magnetic resonance imaging examples of axial slices of: T2 weighted (*left*), diffusion weighted imaging (*center*), and region of interest drawn on an attenuation diffusion coefficient map (*right*)

After generating the ADC maps, a region of interest (ROI) was manually drawn by one of three experienced radiologists (F.R.L., A.K.B.J.N., R.R.R.) on a single DWI slice that showed the lesion at its maximum diameter, using axial FSE T2WI for guidance. PACS software (PixViewer, Viewer MPR - PIXEON) then calculated the ADC minimum (ADC_min_), mean (ADC_mean_), maximum (ADC_max_) and standard deviation of the ADC values (ADC_dev_) (×10^−3^ mm^2^/s) of the chosen region (Fig. 1).

### Statistical considerations

The Mann Whitney test was used to compare ADC values of clinical-pathological and treatment-related factors including: FIGO stage (I/II vs III/IV), histology (squamous vs non-squamous), tumor grade (1-2 vs 3), lymph node status (N+ vs N-), parametrial invasion (yes vs no), vaginal invasion (yes vs no), rectal/bladder invasion (yes vs no), Gross Tumor Volume (GTV) (greater than or less than the median), radiation dose to the primary tumor expressed as an EQD2 (greater than or less than the median) and usage of chemotherapy (yes vs no).

ROC curves were built in order to choose a cutoff value for ADC variables. Confirmatory multivariate Cox model analysis was used to test ADC values and associations with DFS and DSS. These models were adjusted by FIGO stage. LRC was evaluated by confirmatory logistic regression using the GTV as the adjustable variable. Three-year survival rates (DFS and DSS) were estimated according to Kaplan Meyer method. Significance level was set at 5 % for all statistics.

### Availability of data

The data that support our findings will not be shared due to patient privacy issues and the lack of written consent form signed by patients (retrospective study).

## Results

### Correlations between imaging parameters and baseline clinical features

Of the 66 women included in the analysis, 44 had FIGO stage I-II disease while 22 had stage III-IV disease. Seventy-one percent had well or moderately differentiated disease and 82 % had squamous cell cancer. Additional patient details are presented in Table 1.

**Table 1:** Patient and treatment characteristics

	*n* (%)
Number of patients	66
Median age at diagnosis (range)	51.8 (23.3–90.1)
Histology	Squamous: 54 (82 %)
	Adenocarcinoma: 7 (11 %)
	Adenosquamous: 5 (8 %)
Grade (differentiation)	Well: 4 (6 %)
	Moderate: 43 (65 %)
	Poor: 19 (29 %)
FIGO Stage (2009)	IB1-2: 2 (3 %)
	IIA1-IIB: 42 (64 %)
	IIIA-B: 16 (24 %)
	IVA-B: 6 (9 %)
Median External beam radiotherapy dose (range)	44.92 Gy (39.6–59.4)
Median HDR Brachytherapy dose (range)	27.05 Gy (21–28)
Median Total external beam and brachytherapy dose as an EQD2 (range)	82.2 Gy (74–83.9)
Concurrent chemotherapy	58 (88 %)

Table 2 shows comparisons between baseline clinical features and different ADC values. Women with FIGO stage III-IV disease had significantly higher mean ADC_max_ values compared with stage I-II (1.8 vs. 1.5, *p* = 0.007). Patients with imaging defined positive nodes also had significantly higher mean ADC_max_ values compared with lymph node negative ones (2.0 vs. 1.6, *p* = 0.029). No other significant correlations were seen.

**Table 2:** Mean ADC values according to tumor stage, lymph node involvement, and MRI assessed disease extent

	ADC_min_	ADC_mean_	ADC_max_	ADC_deviation_
*FIGO Stage*	(*p* = 0.077)	(*p* = 0.227)	(*p* = 0.007)	(*p* = 0.070)
I-II (*n* = 44, being 22 with positive nodes)	0.375	0.855	1.485	0.224
III-IV (*n* = 22, being 18 with positive nodes)	0.267	0.901	1.806	0.256
*Lymph node*	(*p* = 0.902)	(*p* = 0.092)	(*p* = 0.029)	(*p* = 0.154)
Positive (*n* = 40)	0.323	0.959	1.995	0.232
Negative (*n* = 26)	0.341	0.861	1.551	0.197
*Parametrial Invasion*	(*p* = 0.680)	(*p* = 0.810)	(*p* = 0.081)	(*p* = 0.492)
Present (*n* = 59)	0.332	0.867	1.618	0.223
Absent (*n* = 7)	0.404	0.898	1.371	0.177
*Vaginal Invasion*	(*p* = 0.190)	(*p* = 0.252)	(*p* = 0.203)	(*p* = 0.887)
Present (*n* = 47)	0.311	0.848	1.624	0.198
Absent (*n* = 19)	0.410	0.925	1.513	0.267
*Adjacent structure invasion (rectum or bladder)*	(*p* = 0.683)	(*p* = 0.184)	(*p* = 0.301)	(*p* = 0.829)
Present (*n* = 21)	0.353	0.904	1.702	0.192
Absent (*n* = 45)	0.339	0.854	1.542	0.231
*Gross tumor volume (cc)* ^a^	(*p* = 0.172)	(*p* = 0.886)	(*p* = 0.147)	(*p* = 0.468)
> 114.48	0.295	0.862	1.674	0.237
< 114.48	0.383	0.877	1.508	0.206

^a^Calculated by multiplication of the tumor measures (left-right, anterior-posterior, cranial-caudal)

### Treatment outcomes

After a median follow up of 32 months (range 5–43), 11 patients (17 %) developed recurrent disease (from whom three were still alive by the time of the analysis) with a median time to recurrence of 9 months (range 5–39). Two patients developed pelvic recurrence only (one an in-field recurrence in the cervix and one in a left external iliac lymph node), five developed distant metastasis only, and four developed both pelvic/distant disease recurrence. For these four patients, the pelvic component of failure included: two in the cervix only and two in the cervix and pelvic lymph nodes. Six out of eight women did not receive concurrent chemo (and were free of disease by the time of the analysis.

There have been a total of nine deaths in the 66 women with a median time to death of 13 months (range 9–32). Eight patients (12 % of the total 66) have died from cervical cancer (they presented cancer recurrence) and one patient died from a pulmonary embolus who had no evidence of disease at the time of death. Baseline clinical features of the eight patients who died from cervical cancer include: median age 57 (range 36–74), 6/8 SCC, 8/8 moderate/poorly differentiated, 6/8 FIGO stage III-IV, 6/8 received chemotherapy, median GTV volume 154 cc, and median EQD2 of external beam and brachytherapy was 82 Gy (range 74–83.9).

The 3-year LRC and DFS for the entire group were 89.3 and 84.8 %, respectively. The 3-year DSS was 87.5 %. Table 3 shows the univariate analysis for correlation between clinical characteristics with DFS and DSS.

**Table 3:** Univariate analysis for disease specific survival and disease free survival

			Disease specific survival		Disease free survival	
Variable	Category	*n*	3-y DSS	*p*-value	3-y DFS	*p*-value
FIGO	I / II	44	95.3	0.007	90.9	0.018
	III / IV	22	72		72.7	
Lymph node	Positive	40	79.1	0.015	74.9	0.024
	Negative	26	100		100	
Parametrial Invasion	Present	59	85.9	0.304	83	0.816
	Absent	7	100		100	
Vaginal Invasion	Present	47	84.3	0.265	82.9	0.850
	Absent	19	94.7		89.5	
Adjacent Structures Invasion	Present	21	71.1	0.005	69.3	0.015
	Absent	45	95.3		93	
GTV (^a^)	<114.48	41	97.6	0.001	95.1	0.003
	>114.48	25	69.4		68	
ADC_min_ (^a^)	<0.488	*45*	*93.1*	0.060	90.9	0.073
	> 0.488	21	76.2		72.7	
ADC_mean_ (^a^)	< 0.827	25	100	0.017	96	0.037
	> 0.827	41	79.3		77.9	
ADC_max_ (^a^)	< 1.838	51	93.9	0.002	90.2	0.053
	> 1.838	15	64.6		66	
ADC_dev_ (^a^)	< 0.148	16	100	0.088	93.8	0.221
	> 0.148	50	83.2		81.9	

(^a^)Cutoff values were defined by ROC curve analysis

Cutoff points for predicting the analyzed outcomes were chosen by ROC curve analysis for ADC_min_ (0.488 × 10^−3^ mm^2^/s, AUC = 0.57; 95 % CI: 0.33–0.79), ADC_mean_ (0.827 × 10^−3^ mm^2^/s, AUC = 0.72; 95 % CI: 0.56–0.88), ADC_max_ (1.838 × 10^−3^ mm^2^/s, AUC = 0.70; 95 % CI: 0.50–0.90) and ADC_dev_ (0.148 × 10^−3^ mm^2^/s, AUC = 0.60; 95 % CI: 0.41–0.78).

**Fig. 2:**
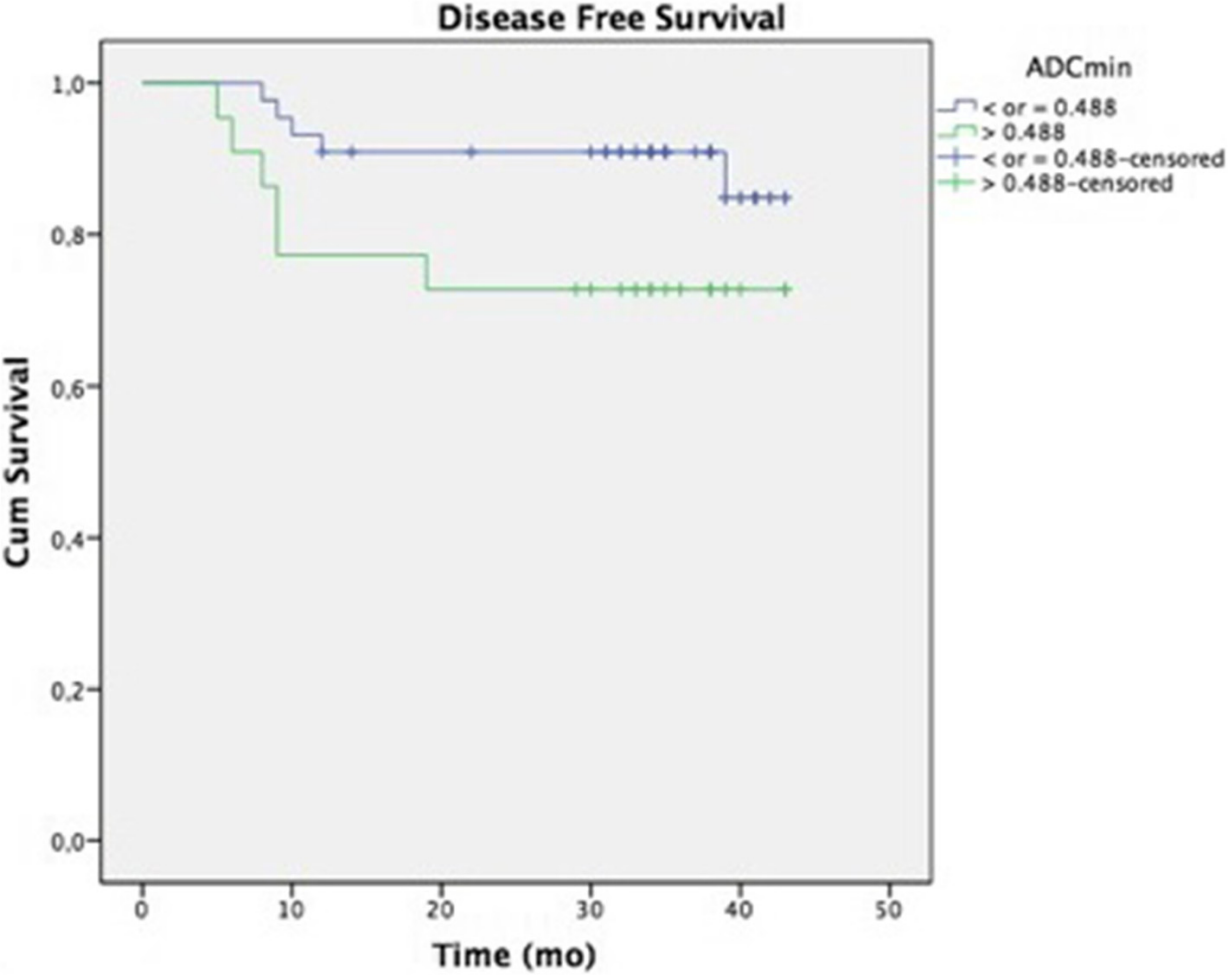
Kaplan Meyer curve for DFS

**Fig. 3:**
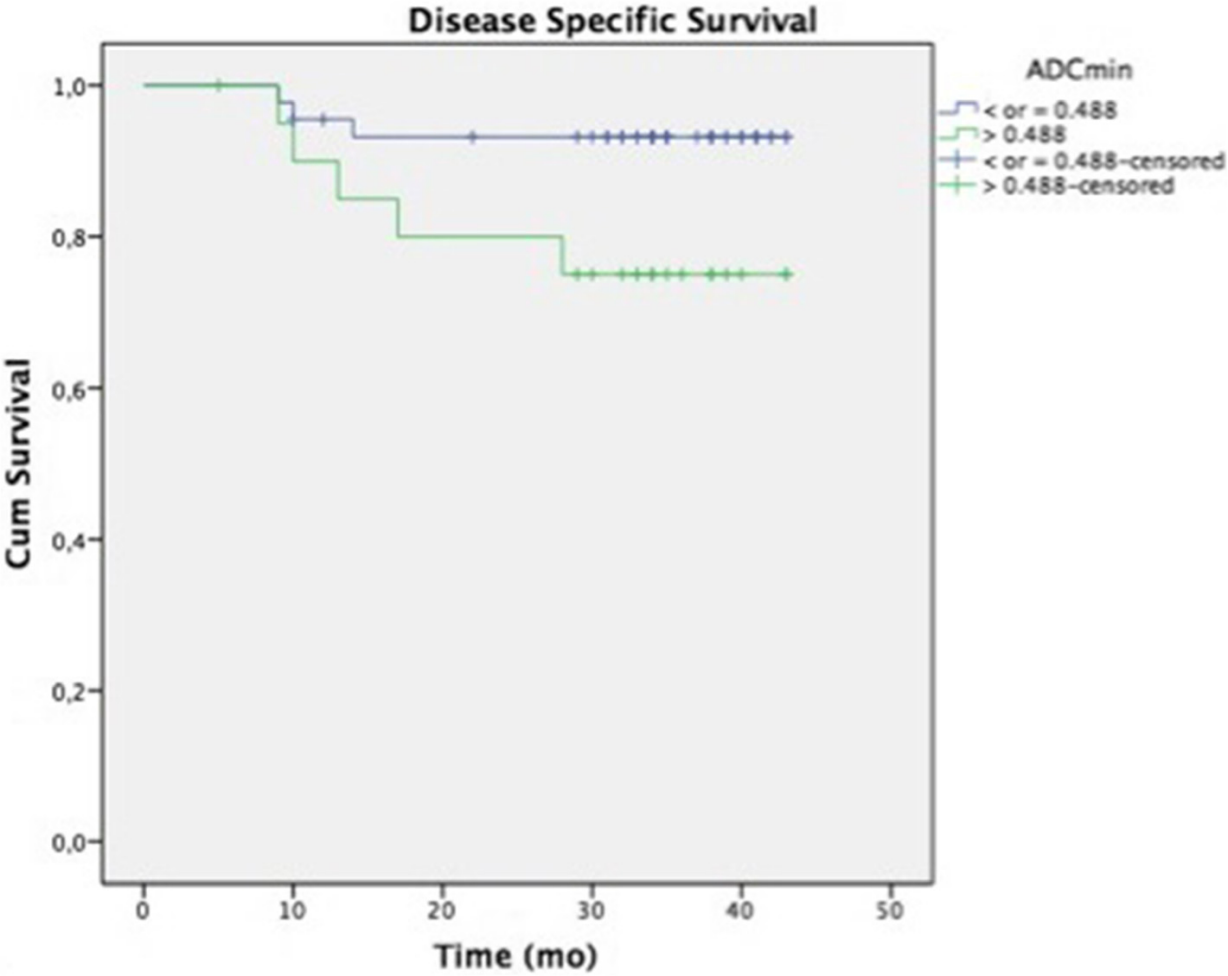
Kaplan Meyer curve for DSS

Tables 4 and 5 show the multivariate analysis for LRC and survival, respectively. No ADC value was correlated with LRC. ADC_min_ higher than the cut off was independently associated with worse DFS (HR = 3.6–95 % CI: 1.09–12.05; *p* = 0.035) and DSS (HR = 4.4–95 % CI: 1.05–18.5; *p* = 0.043). Figures 2 and 3 show Kaplan Meyer curves for DFS and DSS, respectively.

**Table 4:** Multivariate logistic regression models for local regional control

Variables	Category (*1)	*n*	HR (*2)	95 % CI
ADC_min_	< 0.488	45	Ref.	
	> 0.488	21	3.9	0.6–27.7 (*p* = 0.169)
ADC_mean_	< 0.827	25	Ref.	
	> 0.827	41	1.7	0.2–17.7 (*p* = 0.650)
ADC_max_	< 1.838	51	Ref.	
	> 1.838	15	1.3	0.2–10.0 (*p* = 0.771)
ADC_dev_	< 0.148	16	Ref.	
	> 0.148	50	0.9	0.1–9.7 (*p* = 0.934)

*HR* hazard ratio, *CI* confidence interval, *Ref* reference

(*1) Cutoff values were defined by ROC curve analysis

(*2) Each model was adjusted by Gross Tumor Volume (GTV: cutoff value = 114.48)

**Table 5:** Multivariate models for disease specific survival and disease free survival

			Disease specific survival		Disease free survival	
Variable	Category (*1)	*N*	HR (*2)	95 % CI	HR	95 % CI
	< 0.488	45	Ref		Ref	
ADC_min_	> 0.488	21	4.4	1.1–18.5 (*p* = 0.043)	3.6	1.1–12.1 (*p* = 0.035)
	< 0.827	25	Ref		Ref	
ADC_mean_	> 0.827	41	277.3	0.0–2.7 (*p* = 0.963)	4.9	0.6–39.5 (*p* = 0.138)
	< 1.838	51	Ref		Ref	
ADC_max_	> 1.838	15	4.3	1.0–19.3 (*p* = 0.056)	2.1	0.6–7.3 (*p* = 0.255)
	< 0.148	16	Ref		Ref	
ADC_dev_	> 0.148	50	202.5	0.0–2.4 (*p* = 0.969)	2.7	0.4–21.1 (*p* = 0.354)

*HR* hazard ratio, *CI* confidence interval, *Ref* reference

(*1) Cutoff values were defined by ROC curve analysis

(*2) Each model was adjusted by FIGO staging (I/II vs III/IV)

## Discussion

Recent advances in imaging have improved the ability to characterize the full extent of local disease extension, pelvic/para aortic lymph node involvement, and the presence of distant metastasis in cervical cancer patients [11]. Functional information derived from PET/CT like the SUV_max_ can also be prognostic [5–7]. Unfortunately this advance in PET/CT imaging is not readily available in developing countries such as Brazil. MRI imaging is, however, more accessible. MRI has the advantage of providing superior soft tissue anatomy compared with CT, which in turn improves assessment of locoregional disease. In addition, functional MRI sequences have the potential to make MRI more than just an anatomic tool. Areas of interest can be contoured on DWI imagined and be quantified by calculating an ADC value. DWI imaging is also very practical in that it does not require much additional scan time or require intravenous contrast [10, 12–14].

In this study we found that pre-treatment ADC_max_ was significantly correlated with FIGO stage and radiographically enlarged lymph nodes. A recent study from Memorial Sloan Kettering Cancer Center also showed a significant correlation between pretreatment ADC_mean_ with FIGO stage and the presence of positive lymph nodes [15]. However, while their study showed significantly higher ADC_mean_ for earlier staged disease and uninvolved nodes we found exactly the opposite result (higher values of ADC_max_ for higher staged disease and positive nodes). This difference demonstrates some of the challenges of comparing results between studies given non-standardized methods for calculating and reporting ADC results and is discussed further below.

Investigating whether baseline ADC values might be a prognostic imaging biomarker may be more important than a correlation with baseline tumor characteristics. If validated this would give us an opportunity to consider risk adapting patients at the start of their treatment rather than waiting until a recurrence or subjecting all patients to an increased intensity regimen, where only a few might actually benefit. Whether ADC values are prognostic, similar to SUV_max_, is an area of active investigation with existing publications showing both increased and decreased pre-treatment ADC tumor values correlating with clinical outcomes [20–22].

The literature to date using ADC for assessing prognosis has predominantly focused on metrics such as ADC_min_, ADC_max_, ADC_mean_, and ADC percentiles when a histogram-based analysis is used. One recent histogram based analysis includes a recent study of 85 cervical cancer women treated with chemoradiation demonstrating a lower baseline absolute and normalized ADC 95^th^ percentile is associated with shorter disease free survival on multivariate analysis [16]. Other groups have reported on using ADC information gleaned from a single MRI slice. In one retrospective study of 45 cervical cancer women treated with a mix of definitive surgery and chemoradiation, a lower pretreatment ADC_mean_ was predictive of both disease free and overall survival [15]. While these are two of the larger studies published to date looking at correlations between ADC values and clinical outcomes in cervical cancer, there are multiple studies that have been published on this topic with variation in the correlation between ADC values and outcomes. Some studies have correlated higher pre-treatment ADC values with inferior outcomes, while others have correlated lower pre-treatment values with inferior outcomes. These inconsistencies are likely related to multiple factors including: the heterogeneity of the patients, different treatments (surgery vs chemoradiation), various histologies (squamous cell cancer vs adenocarcinoma), use of a single slice region of interests for calculating ADC values which can underestimate the true heterogeneity of the overall tumor, different MRI imaging protocols, retrospective study design, different time points for assessing treatment response, and small patient numbers. These discrepancies point to some of the challenges in comparing data across various studies. Moving forward there needs to be agreed upon imaging and reporting standards so that data can be compared across different institutions. This is not a problem unique to DWI and similar discrepancies have been reported for dynamic contrast enhanced MRI studies in cervical cancer [17].

We looked at standard ADC metrics like the minimum, maximum and mean but also evaluated the ADC_dev_, which has not been previously reported on. ADC_min_ and ADC_max_ represent extreme values that can be very sensitive to tumor composition, i.e., extremely high or extremely low ADC sub-volumes (which could have prognostic value). The fact that ADC_mean_ represents a much larger amount of information (it represents the mean value of all voxels measures including the ADC_min_ and ADC_max_) could explain the observation that it reached significance only in the univariate analysis but didn’t do in the multivariate where only ADC_min_ was significantly associated to outcomes - higher values were correlated with poorer DSS and DFS. A number of studies have linked ADC values to therapy outcomes, with most of them showing that tumors with higher values respond less favorably to therapy [23–27] . Mechanistically this may be explained by the presence of microscopic and macroscopic tumoral necrosis, which can increase ADC values and is linked to poorer outcomes [18, 28]. Our data is in contrast however to Nakamura et al. [29] who analyzed the combination of ADC_min_ and SUV_max_ in 66 women with cervical cancer. Women with lower ADC_min_ showed decreased OS compared to those with the highest values. The fact that we found exactly the opposite in our study (highest values of ADC_min_ predicting worse DSS) only exemplifies the difficulties in interpreting this data without standardized reporting.

When looking at the makeup of the patients who died from cervical cancer one can see that although the majority had advanced features (FIGO III/IV disease, moderate/poorly differentiated SCC, and large GTV volumes) that there were many patients with similar features who had positive outcomes. This emphasizes the limitations of our current risk stratification schemes that focus on clinical and pathologic features without integrating information about the biology of the tumors. While the underlying biology responsible for variations in ADC values in cervical cancer is not known it does provide functional information that is currently not incorporated into our standard risk stratification tools. Given that the dominant pattern of failure in our cohort included a component of distant failure (nine out of 11 cases) it is critical that we identify women at high risk of distant failure as early on in the natural history of their disease as possible in an effort to improve their outcomes. With additional data it’s possible that information gleaned from functional imaging could help identify high risk populations either independently or synergistically with our current clinically based stratification.

There are some weaknesses of our study which include its retrospective design. This contributes to differences in the timing and method of assessment of clinical response as well as the different b-values used for the DWI studies. Variation in b-values occurred due to the adoption of different imaging protocols over time. A study published by Hoogendam et al. however reported that changing the tested b-value combinations did not influence the ADC-based differentiation of benign tissue from malignant tissue and so it is not clear if this impacted the results of this study [30]. Also, we limited the number of adjusted variables in the confirmatory multivariate model in order to avoid an over fitting due to the relative small number of events [31]. Hence, we decided to use well-known prognostic factors as adjusted variables such as FIGO stage for DSS and DFS and GTV for LRC. Moreover, patients were treated using 2-dimmentional brachytherapy and did not have their enlarged lymph nodes boosted. This might have impacted the patterns of failure and ultimate treatment outcomes as has been suggested by the improved outcomes using 3-dimmentional image guided brachytherapy data, however, the local failure rates in this series are low and the predominant failure pattern was distant which is in line with more modern image guided outcomes.

These findings need to be validated in a prospective setting and we have already open a clinical trial measuring ADC values at baseline, mid-treatment, and 3 months post-treatment in patients being treated with chemoradiation for cervical cancer. Ultimately a prospective trial will help determine whether baseline or mid-treatment MRI features, as has been suggested by others are independent predictors of outcomes and whether this could be used for selecting patients that may benefit from escalated treatment [32, 33].

## Conclusions

Pre-treatment ADC_max_ measured in the primary tumor may be associated with FIGO stage and lymph node status. Higher pre-treatment ADC_min_ measured in the primary tumor of cervical cancer might predict worse disease free survival and disease specific survival in patients treated with definitive chemoradiation. Prospective validation of these findings is currently ongoing.

## Abbreviations

ADC, apparent diffusion coefficient; ADCdev, standard deviation of the ADC; ADCmax, maximum value of ADC; ADCmean, mean value of ADC; ADCmin, minimum value of ADC; AUC, area under curve; CI, confidence interval; DFS, disease free survival; DSS, disease specific survival; DW, diffusion weighted; HDR, high dose rate; HR, hazard ratio; LRC, local regional control; MRI, magnetic resonance imaging; PET, positron emission tomography; ROC, receiver operating characteristic; ROI, region of interest; SCC, squamous cell carcinoma; SD, standard deviation; SUV, standardized uptake value
